# The Basolateral Amygdala: The Core of a Network for Threat Conditioning, Extinction, and Second-Order Threat Conditioning

**DOI:** 10.3390/biology12101274

**Published:** 2023-09-22

**Authors:** Tayebeh Sepahvand, Kyron D. Power, Tian Qin, Qi Yuan

**Affiliations:** Biomedical Sciences, Faculty of Medicine, Memorial University, St John’s, NL A1B 3V6, Canada; tsepahvand@mun.ca (T.S.); kdp764@mun.ca (K.D.P.); tianq@mun.ca (T.Q.)

**Keywords:** amygdala, threat conditioning, extinction, second-order conditioning, sensory cortex, hippocampus, prefrontal cortex, norepinephrine, dopamine

## Abstract

**Simple Summary:**

Threat conditioning is a process by which animals learn about danger. As such, this process has been extensively studied as a model of how humans develop fears and anxiety, and their related disorders such as post-traumatic stress disorder (PTSD), which affects seven to eight percent of adults at some point in their life. Conversely, threat extinction is essentially the reverse of this process, by which animals adaptively learn that something is no longer dangerous. Threat extinction forms the basis of exposure therapy, a cornerstone in the treatment of such disorders, during which patients face their fears in order to overcome them. So-called higher-order forms of learning such as second-order threat conditioning add layers of complexity to threat conditioning and may thus more closely mimic processes underlying the development of such disorders. While the amygdala has long been thought to be at the center of such processes, being activated and suppressed during threat conditioning and extinction, respectively, in reality a complex network of structures governs these forms of learning. While such networks are not proposed to hold all the answers to our understanding of the development and treatment of such disorders, they are an important piece of the puzzle, and are thus reviewed here. Recent advances are presenting new areas of exploration into the brain-based mechanisms of such forms of learning and may ultimately inform the treatment of fear- and anxiety-related disorders.

**Abstract:**

Threat conditioning, extinction, and second-order threat conditioning studied in animal models provide insight into the brain-based mechanisms of fear- and anxiety-related disorders and their treatment. Much attention has been paid to the role of the basolateral amygdala (BLA) in such processes, an overview of which is presented in this review. More recent evidence suggests that the BLA serves as the core of a greater network of structures in these forms of learning, including associative and sensory cortices. The BLA is importantly regulated by hippocampal and prefrontal inputs, as well as by the catecholaminergic neuromodulators, norepinephrine and dopamine, that may provide important prediction-error or learning signals for these forms of learning. The sensory cortices may be required for the long-term storage of threat memories. As such, future research may further investigate the potential of the sensory cortices for the long-term storage of extinction and second-order conditioning memories.

## 1. Introduction

**Figure 1:**
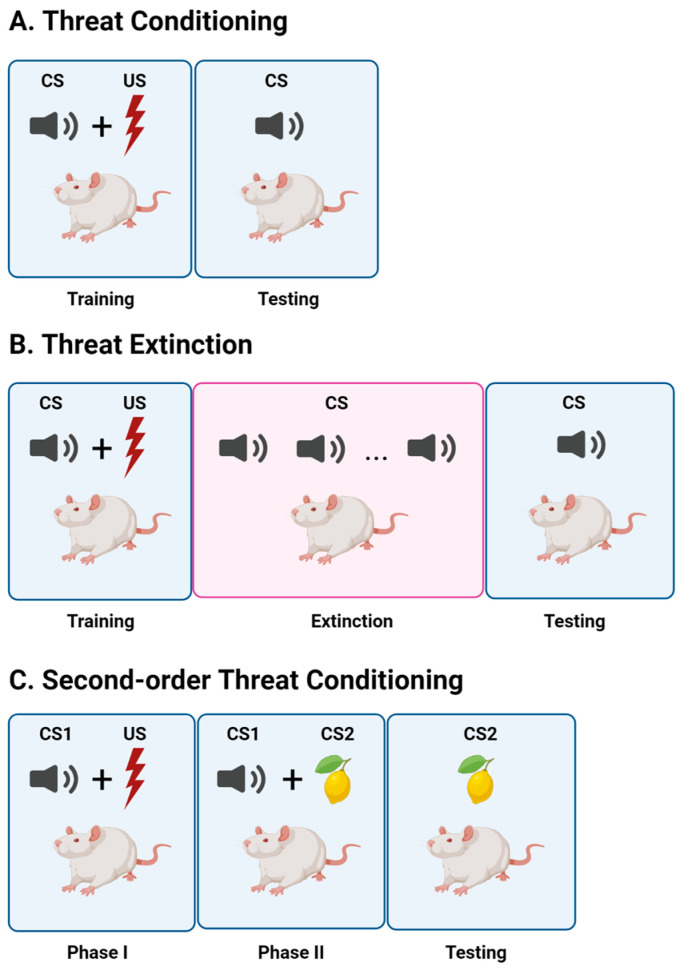
Threat conditioning, extinction, and second-order threat (SOC) conditioning. (**A**) During threat conditioning, an initially neutral conditioned stimulus (CS) is paired with an innately aversive unconditioned stimulus (US), typically a foot shock. Conditioned responses (CR), such as freezing, may then be measured in response to the CS. (**B**) To establish threat extinction, following threat conditioning, presentations of CS alone trigger formation of a CS–no US association such that the CS no longer elicits a defensive CR. (**C**) To establish SOC, after initial threat conditioning with CS1 in phase one, an additional neutral stimulus (CS2) is paired with CS1 in phase two. During testing, CS2 elicits a defensive CR via indirect association with the US.

Currently, more than 300 million people are living with fear- and anxiety-related disorders (World Health Organization). Threat conditioning has been studied extensively due to its proposed contribution to such disorders [1]. In Pavlovian threat conditioning, an aversive unconditioned stimulus (US), typically a foot shock, is paired with a neutral conditioned stimulus (CS), such as an auditory tone (Figure 1A). After threat conditioning, the tone CS becomes capable of eliciting a conditioned response (CR) such as defensive freezing behavior. Also originally described by Pavlov is the process of extinction by which the association between the CS and US is unlearned or suppressed with repeated exposure to the CS alone [2,3]. Threat extinction involves the repeated presentation of the CS alone, resulting in diminished freezing responses to the CS (Figure 1B), and forms the basis of exposure therapy [4], in which patients learn that the things they fear no longer pose a threat to their well-being. Higher-order forms of conditioning, such as second-order conditioning, also defined by Pavlov although relatively less studied, offer additional layers of complexity and may thus more closely mimic certain aspects of psychiatric conditions such as fear and anxiety-related disorders. In SOC, the pairing of a neutral stimulus (CS2, such as a visual cue or odor) with CS1 (such as a tone) that has acquired negative valence through its association with a US (such as a foot shock) confers this negative valence to CS2, such that CS2 can elicit defensive responses (Figure 1C). The basolateral amygdala (BLA) has been and remains a centerpiece in our understanding of threat learning and extinction and as such will be a key area of discussion for the purpose of this review. However, recent evidence suggests that threat memories are more widely distributed beyond the BLA than previously conceived. Although such models lack the complexity of human fear memories, they allow for the study of distributed memory networks which likely contribute to the predictive concepts or schema thought to underlie human emotions such as fear and anxiety as well as their disorders [5,6,7]. As such, the study of such animal models is paramount for our understanding of the nature of fear and anxiety and their related disorders and thus may inform their treatment.

## 2. Threat Conditioning

### 2.1. The Role of the Basolateral Amygdala in Threat Conditioning

**Figure 2:**
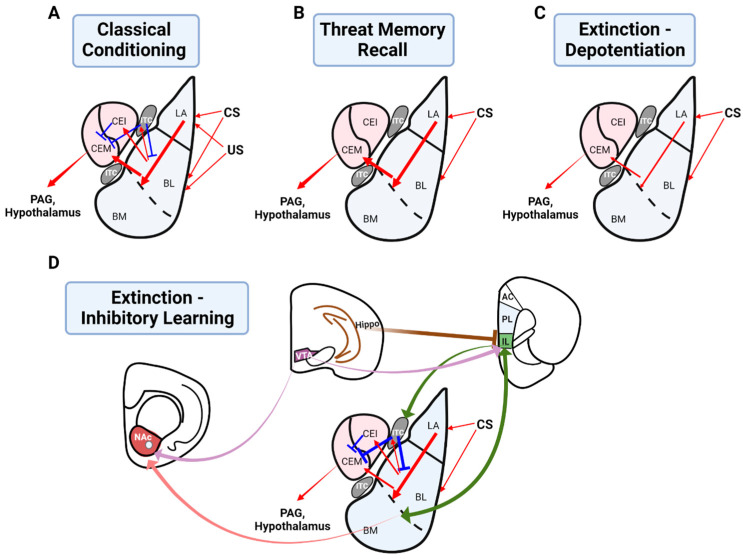
A simplified depiction of the circuitry underlying threat conditioning and extinction. (**A**) The LA connects to the CeA directly or by way of the BA (containing lateral (BL) and medial (BM) divisions) or ITCs. The BA similarly connects to the CeA directly or by way of ITCs. The medial division of the CeA (CeM) projects to downstream structures such as the hypothalamus and PAG to control autonomic and defensive responses. CS and US convergence in the BLA leads to synaptic changes via a complex interplay of excitation and inhibition within the amygdala. Red lines indicate the amygdala outflow tract, blue lines indicate inhibition of amygdala outflow. (**B**) During threat memory recall, potentiated BLA synapses allow CS inputs to drive output via the CeA, triggering a CR. (**C**) The depotentiation model of threat extinction proposes that extinction involves a depotentiation of the synapses potentiated by threat conditioning in the BLA such that the CS can no longer drive CeA activity. Thin lines indicate a weakening of synapses in the BLA. (**D**) An inhibitory learning model suggests that extinction learning forms an inhibitory extinction memory with crucial nodes at IL inputs to the BA and ITCs. Also involved are VTA-NAc, VTA-IL, and BLA-NAc projections, as well as hippocampal control of the IL, which may confer context dependency. As indicated by thick lines, the initial threat memory is not depotentiated but overridden by the extinction memory such that the BLA cannot drive CeA activity.

The lateral (LA) and basal amygdala (BA), collectively referred to as the BLA, are considered the main input sites of the amygdala, receiving sensory information including that regarding auditory, olfactory, and visual stimuli via thalamic and cortical routes [8,9] as well as information regarding unconditioned stimuli such as foot shocks via the somatosensory system and brainstem. As such, the BLA is well-situated anatomically to associate stimuli with aversive events, a brief overview of which is presented here (see reviews [1,10,11]). The central amygdala (CeA) comprises lateral (CeL) and medial (CeM) subdivisions, the latter of which is considered the main output site of the amygdala. The LA projects to the CeA via direct connections to the CeL, indirectly via BA projections to the CeM [11], as well as via inhibitory intercalated cells (ITCs) [12], which in turn project to the CeA [13]. The BA also influences the CeA via the excitation of ITCs, which send GABAergic inputs to the CeA [14]. Output from the CeM is mainly GABAergic and projects to the hypothalamus and brainstem (e.g., periaqueductal grey (PAG)) to mediate autonomic responses and defensive behavior [15]. Considerable evidence suggests that threat conditioning is mediated by long-term potentiation (LTP) in the BLA (see reviews [1,10,16]). The coincident activation of CS and US ensembles in the BLA is known to induce synaptic plasticity at cortical and thalamic synapses relaying CS information, thus potentiating BLA responses to the CS [17] (Figure 2A). Thus, following threat conditioning in which a CS is paired with a US, the CS itself comes to trigger defensive behavior (e.g., freezing) through potentiated CS-BLA synapses and enhanced output from the CeM [18,19] (Figure 2B). More recently, Hagihara et al. [20] showed that distinct ITC neurons exerted opposing functions during threat acquisition and extinction. Furthermore, threat conditioning is thought to suppress feedforward inhibition onto principle LA neurons, allowing for LTP at excitatory thalamo-LA synapses [21,22].

The acquisition of threat learning is thought to depend on postsynaptic NMDA-receptor (NMDAR) activation at relevant synapses. For instance, the infusion of broad-spectrum NMDAR antagonists disrupts the acquisition of threat learning [23,24,25]. Increased calcium influx through NMDARs leads to the phosphorylation of CaMKII, which is known to increase in dendritic spines of the LA after threat conditioning [26,27]. Resultant changes in synaptic strength are thought to be mediated in part by post-synaptic AMPA-receptor (AMPAR) insertion. Threat conditioning induces an increase in calcium-permeable AMPARs (CP-AMPARs) which peaks at 24 h post-conditioning, corresponding with a labile reconsolidation window for the updating of threat memories [28,29]. The consolidation of threat learning in the LA further requires protein synthesis [30]. Epigenetic mechanisms such as histone acetylation and DNA methylation have also been implicated in threat conditioning in the LA. For example, auditory threat conditioning promotes an increase of acetylation of histone H3 in the LA following threat memory retrieval, and inhibition of histone deacetylase (HDAC) in the LA enhances auditory threat memory consolidation [31]. Further, inhibition of DNA methyltransferase (DNMT) activity in the LA impairs consolidation of auditory threat conditioning [31]. Thus, epigenetic mechanisms are likely important for synaptic plasticity and thus threat memory consolidation in the LA. As such, epigenetic changes, which are thought to occur relatively early during learning and in a reversible manner, may represent a novel therapeutic window insofar as the processes they support contribute to fear and anxiety-related concepts or schema.

### 2.2. Neuromodulation of the Basolateral Amygdala in Threat Conditioning

Hebbian processes in the LA may be on their own insufficient to produce the lasting synaptic changes underlying threat conditioning, thus additionally requiring neuromodulation from the brainstem arousal system. For instance, the neuromodulator norepinephrine (NE) is sourced to the BLA from the brainstem locus coeruleus (LC) [32,33] and is required in the LA during the acquisition of threat conditioning [34,35]. The effect of NE in the LA may synergize with Hebbian processes occurring in principle LA neurons and may further gate LTP via a reduction of GABAergic inhibition onto LA neurons [22] known to interfere with the acquisition of threat learning [36,37]. Output via the CeA is known to recruit the LC for the facilitation of amygdala processing [38]. Auditory threat conditioning results in enhanced firing of BLA neurons, which is prevented by systemic β-adrenoceptor antagonism and augmented by chemogenetic activation of the LC [39]. Optogenetic stimulation of LC-BLA projectors enhances threat conditioning [38,40]. Owing to a recent conceptualization of the LC as being heterogeneous in structure and function, LC enhancement of BLA processing may also occur in a modular manner [41]. Similarly, the CeA is known to innervate the dopaminergic ventral tegmental area (VTA) [42], the concentration of DA in the BLA is known to increase with arousal [43], and dopaminergic VTA neurons respond to both the US and CS following threat conditioning [44]. Infusion of the dopamine D1 receptor antagonist SCH 23,390 into the BLA impaired threat learning and memory [45]. The suppression of feedforward inhibition allowing for LTP in the LA during threat conditioning is also gated by dopamine via D2 receptors [21,46]. It has recently been shown that the majority of VTA-BLA projectors are dopaminergic and that optogenetically silencing this pathway impairs threat memory [47]. Thus, it is likely that a complex interplay of Hebbian processes with neuromodulation is required for threat conditioning in the BLA.

### 2.3. Greater Circuitry Involved in Threat Conditioning

Even though the BLA has been at the forefront of threat conditioning research, threat memories have been more recently conceptualized to be encoded and stored in a distributed network of structures, including prefrontal, orbitofrontal, hippocampal, and the more recently debated sensory cortices [48,49,50,51]. It has been postulated that an interaction between the prelimbic (PL) prefrontal cortex (PFC) and BLA is important for the acquisition of threat conditioning, while the infralimbic (IL) PFC is necessary for its extinction [11,52]. A balance of activities between the PL and IL appears to be important for the expression and suppression of threat memories [53,54]. Recent research suggests that distinct hippocampal ensembles are differentially activated during threat conditioning and extinction [55]. An interaction between the BLA and hippocampus is likely particularly important for contextual threat learning [56]. Silencing the BLA blocks the acquisition and extinction of contextual threat conditioning [57,58].

While the BLA is initially critical for the encoding of threat memories for short-term storage, the long-term storage and expression of threat memories has been located in the sensory cortices, and especially secondary/associative sensory cortices in both human and animal studies [59,60,61,62,63,64,65]. For example, the roles of the auditory and olfactory piriform cortices have been reviewed extensively [49,66,67]. Both auditory and piriform cortices have extensive mutual connections with the BLA [8,68,69,70]. Synaptic plasticity in the forms of LTP or dendritic spine remodeling have been reported in BLA-cortical projections as well as the cortico-BLA inputs [51,68,71,72,73]. Plasticity in the pathway from the LA to layer V of the auditory cortex was reported to contribute to the long-term storage of threat memories [68]. Threat conditioning alters receptive field tuning in the sensory cortices and reshapes cortical firing patterns through altered excitation and inhibition [62,74,75]. Threat conditioning is also known to enhance functional connectivity between the BLA and sensory cortices [63,76]. However, it remains undetermined whether the sensory cortices merely encode the physical identity and salience of sensory stimuli or if they may also encode valence. Some recent studies support the latter. For example, aversive stimuli by themselves can trigger or modify the neuronal activity of the auditory cortex [9,77,78], suggesting that the sensory cortices have the capacity to directly associate CS with US. Both the amygdala and sensory cortices display learning-evoked changes, however, with different temporal profiles [61,79]. Neurons in the primary auditory [80,81,82] and piriform cortices [83] can encode different valences. The relationship between the BLA and sensory cortices in threat conditioning appears to be similar to that of the hippocampus and cortex for spatial and episodic learning. Initially critical for memory encoding, the hippocampus gradually becomes less important over time when remote memory is stored within a distributed cortical network [61,62,84,85]. The parallel storage of threat memories in both subcortical and cortical territories would confer fundamental survival advantages to the animal [66,86] and may pose a challenge to therapeutic intervention.

## 3. Threat Extinction

### 3.1. The Role of the Basolateral Amygdala in Threat Extinction

The first line of evidence suggesting the involvement of the BLA in threat extinction came from electrophysiological recording studies showing reduced amygdala (especially in the CeA) activity in response to the CS following extinction training, correlating with reduced defensive responses to the CS [87,88]. Falls et al. [89] first demonstrated that NMDAR blockade in the amygdala impaired threat extinction. This has been replicated by numerous other experiments [57,90,91,92,93], suggesting that NMDAR-dependent synaptic plasticity in the BLA underlies threat memory extinction. Two dominant theories have been proposed to explain extinction learning. A depotentiation model proposes that a depotentiation of the synapses initially involved in threat conditioning in the BLA underlies threat extinction [94,95]. At the synaptic level, after extinction learning, potentiated CS synapses in the BLA undergo depotentiation, reducing BLA output to the CEA. Subsequently, CeA output is reduced, thus lessening the defensive response (Figure 2C). Inhibitory LTP may also contribute to extinction learning in the BLA [96]. An inhibitory learning model of extinction suggests that extinction learning forms an extinction memory which competes with and suppresses the original memory trace [14,97,98] (Figure 2D). For instance, several studies have demonstrated persistent CS-induced neuronal responses in the BLA following extinction [99,100], consistent with the idea that the original memory trace persists and is simply masked by extinction learning. A recent study using Fos-CreER mice showed that Fos-labeled CS-responsive cells exhibited persistent potentiation following extinction training, although optogenetically induced depotentiation of LA synapses suppressed conditioned threat learning [99]. The fact that conditioned threat and fear responses can relapse is interpreted to imply the existence of an extinction memory which competes with and suppresses the original memory trace laid down by initial threat conditioning [52]. For instance, spontaneous recovery, reinstatement, and renewal refer to the post-extinction relapse of defensive responses after the passage of time, presentation of US, or introduction to a context other than that of extinction learning, such as the initial conditioning context, respectively [101]. Furthermore, evidence suggests that depotentiation and inhibitory learning mechanisms may work together for extinction learning [102,103]. A brief overview of the mechanisms of extinction learning follows (see also reviews [1,104]).

#### 3.1.1. Depotentiation of the Basolateral Amygdala as a Mechanism of Extinction Learning

A conditioned threat memory can be erased by depotentiating synapses using a NMDAR-dependent long-term depotentiation (LTD) protocol (paired-pulse low-frequency stimulation, pp-LFS) [105], and subsequent repotentiation with an LTP protocol can reinstate the threat memory, demonstrating the close relationship between associative LTP in the LA and threat memory formation and supporting depotentiation as a mechanism for threat extinction. Additional evidence supports the depotentiation theory. Kim et al. [95] reported that extinction training reversed synaptic potentiation observed at thalamo-LA synapses induced by auditory threat conditioning to baseline levels, and excluded the ex vivo depotentiation that was normally observed in naïve brain slices. Depotentiation of the thalamo-LA synapses was paralleled by a decrease in AMPAR-subunit surface membrane expression following extinction. Additionally, blocking AMPAR-endocytosis with a glutamate receptor (GluR)-2-derived peptide during training prevented extinction. Similarly, cortical input synapses onto the LA known to exhibit LTP [94] can undergo reversal of LTP with ex vivo pp-LFS, and extinction training excludes pp-LFS-induced depotentiation. A presynaptic mechanism that was dependent on group 1 metabotropic GluR activation, but not AMPAR-internalization, corresponded with pp-LFS-induced cortico-LA depotentiation. Consistent with the depotentiation model, extinction also reverses other biochemical processes that result from threat conditioning, such as increased Akt phosphorylation in the BLA [106]. Lin et al. showed that LFS of cortico-LA inputs induced NMDAR- and calcineurin-dependent depotentiation of LTP and attenuation of threat-potentiated startle responses [107,108]. The LFS also attenuated Akt and mitogen-activated protein kinase (MAPK) expression, molecules that were elevated following threat conditioning.

#### 3.1.2. Inhibitory Plasticity in the Basolateral Amygdala Underlying Extinction Learning

Alternately, an inhibitory intra-BLA model suggests that a form of synaptic plasticity termed inhibitory LTP, of principal BLA neurons, may occur during extinction. High frequency LA stimulation induces NMDAR-dependent LTP of inhibitory synaptic currents recorded from BLA neurons [96]. Additionally, structural plasticity of GABAergic synapses onto principal neurons of the BLA has been reported. Using TetTag mice to label c-Fos activity and zif268 protein as a second activity marker, Trouche et al. [109] reported that extinction learning silenced a subset of original threat conditioning BA ensembles, and silenced BA neurons exhibited perisomatic inhibitory synapse remodeling such as increased GAD67 expression. Consistent with inhibitory synapse remodeling, levels of the GABA_A_ receptor clustering protein gephyrin decrease following the acquisition of threat conditioning but increase following threat extinction training [110].

### 3.2. Greater Circuitry Involved in Threat Extinction: An Inhibitory Learning Model

#### 3.2.1. The Infralimbic Cortex in Threat Extinction

As alluded to, a key component of the inhibitory learning model for threat extinction is the IL, which has reciprocal connections with the amygdala [98,111,112]. IL neurons exhibit increased excitability following extinction [97,113,114], and silencing the IL via infusion of the GABA_A_ agonist muscimol impairs threat extinction [115]. NMDAR activation and NMDAR-dependent burst firing in the IL are required for the consolidation of threat extinction [114,116]. Threat extinction induces increased excitability and AMPA/NMDA ratios in layer V neurons of the IL [117]. Another study showed that the firing of IL neurons to the CS was closely correlated with successful extinction [113]. IL neurons were relatively quiescent to a non-extinguished CS but became responsive to the CS following extinction learning. Pairing the CS with electrical stimulation of the IL elicited low freezing in rats that had not been extinguished. Further, PKA-dependent transcription and translation in the IL are necessary for extinction [118]. These studies thus demonstrate the critical role of IL neuronal activity in successful extinction learning.

#### 3.2.2. An Interaction between the Infralimbic Cortex and Basolateral Amygdala for Threat Extinction

Extensive research has supported the notion that axonal projections from the IL to BLA [14,97,98] constitute a critical inhibitory pathway during extinction. IL activation inhibits the expression of threat conditioning by reducing outflow from the amygdala via an interaction with local inhibitory neurons in the BLA [98]. Furthermore, evidence suggests that distinct threat and extinction neurons in the BA are differentially involved in threat conditioning and extinction circuitry, facilitating and impairing CeA output, respectively [11,119]. Furthermore, LTP has been discovered at both cortical and thalamic inputs to BLA interneurons [120,121,122]. Given that the LTP of thalamo-interneuron synapses is NMDAR-dependent [120], and that NMDAR blockade in the amygdala prevents threat extinction [99,123], it is likely that LTP at these synapses plays a role in threat extinction. The BLA, on the other hand, impacts IL activity through a complex interplay of direct excitation and feedforward inhibition [112]. The consolidation of threat extinction memory is dependent on coordinated signaling in both the BLA and medial PFC (mPFC). Immediate early genes c-Fos and zif268 in both the mPFC and BLA are activated by threat extinction, which is prevented during the spontaneous recovery of defensive responses [119]. The long-term storage of extinction memory is dependent on protein synthesis in the IL [124]. However, conflicting results have been reported regarding the protein synthesis requirement for threat extinction in the BLA. Plasticity in the BLA has been shown to be important for threat extinction [52,89]. The protein synthesis inhibitor anisomycin blocked threat extinction, as well as extinction-induced calcineurin activation [106]. PI3 kinase and MAPK inhibitors also blocked threat extinction. In contrast, Duvarci et al. [125] used a long CS exposure protocol and showed that anisomycin in the BLA left threat extinction intact. Differences in results regarding the protein synthesis requirement in the BLA for threat extinction may be due to differences in extinction protocols (repeated CS vs. single, long exposure of CS).

#### 3.2.3. The Role of Intercalated Cells in Threat Extinction

The potentiation of inhibitory ITCs may be another locus of inhibitory learning [126,127]. A seminal study by Amano et al. [128] showed that threat extinction was associated with increased inhibition of output CeA neurons, which resulted from a potentiation of GABAergic ITC neurons. BLA-ITC synaptic transmission was enhanced following threat extinction, and blocking IL inputs with muscimol prevented potentiation of the BLA-ITC transmission. IL neurons also project directly to the ITCs [97], but see [98]. Thus, IL-mediated reduction of CeM output [126] may occur via ITCs. Since BLA-ITC synapses are capable of bidirectional plasticity [129], these results suggest that convergent inputs from the BLA and IL may induce NMDAR-dependent LTP of ITC neurons which subsequently suppresses CeA output. Micro-circuitry within the CeA and CeL-mediated inhibitory plasticity were also found to be critical components of the extinction circuit [130].

#### 3.2.4. The Role of the Hippocampus in Threat Extinction

The role of hippocampus, which is reciprocally connected to the BLA [131], has been shown to play a role in threat extinction and renewal [132]. Recent research suggests that distinct hippocampal ensembles control extinction and threat memory relapse [55], with one ensemble being activated during threat conditioning and another activated by extinction. Memories of threat conditioning and its extinction are known to become context-dependent following learning [132]. For instance, threat memories are suppressed in the extinction context but can renew in different contexts. Knapska and Maren [133] studied c-Fos activation in various structures in either the extinction context or a different context following extinction learning. Consistent with the finding that distinct ensembles may be involved in threat conditioning and extinction, higher c-Fos expression was observed in the hippocampus in either context compared to non-extinguished groups. Further, optogenetic stimulation and inhibition of the two ensembles produced opposite effects on threat memory expression. Marek et al. [115] reported that activation of ventral hippocampal (vHPC) projections to the IL recruited parvalbumin interneurons and resulted in feedforward inhibition of BLA-projecting principle IL neurons. Selective activation or silencing of vHPC-IL projections impaired threat extinction recall and prevented threat memory renewal, respectively. The authors suggest that vHPC-mediated inhibition of the IL is a critical component for defensive response relapse. Furthermore, in the hippocampus, protein synthesis [134], calcineurin [135], extracellular signal-regulated kinases (Erk) ½ and Mek (MAPK kinase) [136] are necessary for contextual threat extinction. Collectively, BLA-mPFC-vHPC constitutes a tripartite set of circuitry to mediate threat memory extinction and relapse [52], but understanding how exactly context-specificity mediated by the hippocampus regulates mPFC-ITC-BLA circuitry requires further investigation. Synaptic plasticity in the BLA is essential for the encoding of threat extinction and prefrontal control of the BLA is critically involved in threat extinction consolidation and retrieval, while hippocampal control of the IL may mediate threat memory relapse.

### 3.3. Neuromodulation in Threat Extinction

Furthermore, as with initial threat conditioning, LC-NE modulation is critical for extinction. In contrast to the enhancing effect of arousal on the acquisition of threat conditioning in the BLA [35], NE appears to have bidirectional effects on extinction learning that are dependent on the timing of extinction and arousal level [137]. While activation of IL-projectors of the LC promotes extinction learning [41], LC activation is correlated with increased spontaneous firing of the BLA and impairment of extinction learning [39]. Blocking noradrenergic β-adrenoceptors with propranolol either systemically [39,138] or in the BLA [139] rescues an immediate extinction deficit (extinction conducted minutes to hours following threat conditioning, high arousal level), while propranolol infused in the IL impairs delayed extinction retrieval (24 h following conditioning) [118]. Thus, NE dynamically modulates threat conditioning and extinction under different levels of arousal [137]. Recent work also suggests that dopaminergic neurons in the VTA are activated by US-omission early during extinction training [140], resulting in increased DA release to downstream sites such as the nucleus accumbens (NAc) [141,142]. Optogenetic inhibition of the VTA during initial extinction training impairs the acquisition and retrieval of threat extinction [140,142]. Systemic or intracranial infusion of dopamine D2 receptor antagonists in either the IL [143] or NAc [144] impairs threat extinction. The D2 receptor antagonist raclopride also reduces IL neuronal responsiveness to the CS [143]. Furthermore, both BLA-NAc [145] and insular cortex-NAc connections [146] are enhanced following threat extinction training, consistent with the involvement of reward circuitry in extinction and the omission of the US as a reward signal (better than expected) to initiate new learning during extinction.

### 3.4. Experimental Modifications Determine the Strength and Mechanism of Extinction Learning

Finally, extinction protocols themselves can influence the strength of extinction and may determine whether a depotentiation or inhibitory learning mechanism dominates [29,147]. For example, spaced training results in more effective extinction and less spontaneous recovery and renewal compared to massed training [147]. Clem and Huganir [148] further defined a molecular mechanism for threat extinction and a temporal window during which threat memories can be more permanently degraded by CS exposure. As mentioned, threat conditioning induces an increase in CP-AMPARs which peak at 24 h post-conditioning, correlating with a window for reconsolidation [28,29]. Removal of CP-AMPARs during this time reversed threat conditioning-induced synaptic potentiation and erased threat learning permanently. In fact, Monfils et al. [29] first described a “retrieval-extinction” phenomenon for more permanent threat memory erasure. Retrieval-extinction, or extinction within the reconsolidation window, involves a brief re-exposure to the CS 24 hours following initial threat conditioning (retrieval) and within one hour before repeated CS exposure (extinction). This produces a long-term reduction of threat memory, with minimum spontaneous recovery, renewal, and reinstatement [29]. Single-session versus multiple-session extinction training regimes have also been suggested to engage inhibition and depotentiation mechanisms, respectively [149]. Furthermore, whether depotentiation or inhibitory learning is the dominant extinction process may depend on age. Threat memories in juvenile rats can be permanently erased, whereas in older animals, threat memories frequently resurface following extinction [150,151]. This phenomena has been associated with the development of a perineuronal net in the amygdala [150]. Removal of the perineuronal net in adult animals enables erasure of threat memory, similar to a juvenile phenotype. As such, differences in experimental protocol may influence the strength and mechanism of extinction learning.

## 4. Second-Order Threat Conditioning

The first stage in the establishment of SOC is first-order threat conditioning (FOC) (CS1-US). Next, another neutral stimulus is paired to CS1 (CS2-CS1), which acquires its valence [2]. Such models add complexity to FOC and as such may better resemble the complexity of human memories. Both CS1-US and CS2-CS1 pairings are prerequisites for SOC learning to occur, indicating associative learning as opposed to a mere generalization of responding [152]. During CS2-CS1 pairings, CS2 may associate with the CS1 representation [153], the US representation it evokes [152], or a component of the downstream effects of US activation [154]. Experiments which demonstrate the impact of extinguishing responses to CS1 after CS2-CS1 pairings generally support the latter possibilities. For instance, the extinction of CS1 was shown to have no effect on the expression of conditioned responding to CS2 [152,155], leading to the hypothesis that CS2 may directly associate with a US representation or the greater motivational state with which it is associated [156] (see reviews [157,158]). Further, the finding that US habituation does not impact SOC expression suggests that a CS2-motivational state association may underlie SOC, rather than the linking of CS2 with a US representation per se [154]. However, modifications to SOC protocols may instead evoke a chaining-of-associations mechanism. For instance, a unisensory SOC protocol pairing auditory tone pips (CS1) with tone sweeps (CS2) showed that the extinction of CS1 impaired conditioned responding to CS2 [153]. Furthermore, unisensory SOC forms more rapidly than multisensory SOC, which may reflect the formation of a CS2-CS1 link [159]. Similarly, SOC which employs simultaneous CS2-CS1 pairings is sensitive to CS1 extinction [160], which may reflect the formation of CS2-CS1 compound associations or configural units [161].

### Circuit and Molecular Mechanisms of Second-Order Threat Conditioning and Extinction

Indeed, and non-surprisingly given that FOC is a prerequisite for SOC, the BLA appears to be central for this form of learning as well. Consistent with reports demonstrating the preservation of conditioned responses to CS2 after extinguishing responses to CS1 that suggest a more direct CS2-US linking during SOC, the BLA appears to be critical for this association. Intra-BLA infusion of muscimol impaired the acquisition of tone-context SOC [162]. Holmes et al. [163] also showed that activity in the BLA, but not the perirhinal cortex (PRh), is needed during SOC, as pre-CS2-CS1 pairing treatment with musicmol in the BLA, but not the PRh, impaired later defensive responding to CS2, in line with findings from Parkes et al. [164]. Similarly, the retrosplenial cortex (RSC) is not involved in the CS2-CS1 association during visual-auditory SOC, as evidenced by intact freezing to CS2 in pre-training RSC-lesioned rats [165]. Further, post-CS2-CS1 pairing treatment with bupivacaine in the BLA results in impaired freezing to CS2, implicating that BLA activation is necessary for the consolidation of SOC [166]. Similar to threat conditioning, SOC is dependent on NMDAR activity in the BLA [167]. Antagonizing the NR2B subunit of NMDARs in the BLA with ifenprodil before CS2-CS1 pairing impaired subsequent freezing behavior to CS2 [164]. Most recently, Williams-Spooner et al. [168] have shown that the NMDAR-requirement for the acquisition of SOC in the BLA is dependent on prediction-error during this form of learning. If the aversive shock is expected given the context, time, and stimuli present, but is omitted, NMDAR activation in the BLA is required.

Using a series of pharmacological experiments, Lay et al. [166] used a counterbalanced auditory-visual SOC protocol to demonstrate that the CS2-CS1 association in the BLA depends on CaMKII-signaling, DNA-methylation, and gene expression, but not ERK-, MAPK-, PKA-, or PKC signaling or de novo protein synthesis. This lack of requirement for de novo protein synthesis has been interpreted as a handover of the mechanisms used for the consolidation of FOC to that of SOC and has been replicated by Leid et al. [169] and Williams-Spooner et al. [170] in modified SOC protocols. For instance, Leid et al. [169] showed that the lack of a protein synthesis requirement during serial order pairings (e.g., CS2-CS1-US) was contingent upon a previously established CS1-US association, even if the CS1-US association was weak. As with threat conditioning, BLA activation, occurring during CS2-CS1 pairings due to the previous CS1-US association, will likely recruit the LC to bias its own processing, prioritizing formation of the CS2-CS1 association in the BLA. Nader et al. [171] showed that autoreceptor-mediated inhibition of the VTA or intra-BLA antagonism of D1 receptors prior to CS2-CS1 pairings disrupted the acquisition of SOC.

Furthermore, the BLA is critical for the extinction of CS2 memories. Intra-BLA infusion of muscimol prior to extinction trials using CS2 rescues subsequent reduction in freezing to CS2 [162,164]. These results indicate that, like the extinction of FOC, the extinction of SOC relies on activity in the BLA. Additionally, in other forms of higher-order conditioning which primarily require cortical associations such as sensory pre-conditioning [163,172,173], in which the ordering of associations is the reverse of SOC, the BLA is not essential. However, if S2-S1 pairings occur in an aversive context (a paradigm which may be considered similar to SOC), the S2-S1 association forms directly in the BLA [163,172].

## 5. Discussion

The forms of learning discussed here share many mechanisms in common. On a circuitry level, while the BLA is central for all of these forms of learning, the encoding and storage of each appears to depend on a distributed network of structures. The circuitry for threat conditioning and extinction are grossly similar, importantly involving areas such as the BLA, mPFC, and hippocampus. On a molecular level, these forms of learning share many mechanisms, such as their dependency on NMDAR activity. Threat conditioning and its extinction depend on protein synthesis at central hubs within their respective circuitry, a mechanism which is borrowed during SOC.

### 5.1. Prediction Error-Driven Learning

Threat conditioning and its extinction are prediction error-driven forms of learning [174]. Although less studied, this appears to be true of SOC as well. Prediction error signaling has been proposed as a fundamental learning principle [174,175,176], the primary function of which is to update internal models [177] generative of predictions and underlying cognition. Importantly, the release of NE from the LC, like the release of other neuromodulators such as DA, has been described as a teaching, learning, or prediction error signal [35,178,179,180]. Once established, in threat conditioning a CS comes to predict the occurrence of a US; in extinction, its absence; and in SOC, its occurrence more indirectly. As indicated here, activation at specific nodes within the circuitry can evoke activation at other nodes to which they are associated through learning. For instance, SOC, which may be considered an extension of an internal model of threat, allows for a greater repertoire of stimuli to evoke threat processing and defensive responses. In a similar vein, after extinction learning, presentation of the initial conditioning context may trigger a relapse of defensive responding by virtue of its association with a greater threat network. In the various cases in which defensive responses relapse after extinction, a conflict may need to be resolved between antagonistic nodes within threat and extinction circuitry such as those within the mPFC, hippocampus, and BLA. Catecholaminergic neuromodulators, signaling prediction error, would presumably prioritize the processing of prevailing activity. In the case of NE, such prioritization may involve glutamatergic amplification of noradrenergic effects (GANE) or the enhancement of apical amplification, which have been proposed to be involved in context-sensitive modulation under arousal [181,182]. As mentioned, these systems may further bias processing through mechanisms such as modular release and varying levels of arousal. For instance, the LC appears to facilitate threat conditioning and extinction under high and low levels of arousal, respectively [137]. Discrete LC-IL projection engagement may also specifically facilitate extinction circuitry [41]. Furthermore, phasic LC activation has been shown to bias downstream processing towards the reward-related NAc-projecting subpopulation of the BLA [183,184], shown here to be involved in extinction learning, and thus may serve as another source of bias during such learning.

### 5.2. Clinical Relevance

Evidence supports the idea that a homologous set of circuitry in humans and animals including the amygdala, mPFC, and hippocampus is important for threat extinction [52,185,186,187]. The mechanisms of extinction learning have been leveraged to enhance extinction in clinical settings such as exposure therapy. NMDAR activation via systemic or intra-BLA infusion of the partial NMDAR agonist D-cycloserine facilitates threat extinction [188,189]. Furthermore, elevating NMDAR functioning has been shown to be beneficial during clinical exposure therapy [190,191]. In a human fMRI study, administration of the dopamine precursor L-DOPA immediately following extinction training reduced neuronal activity related to threat conditioning (e.g., amygdala, posterior hypothalamic–pituitary–adrenal (HPA) axis) and enhanced activity in areas related to extinction retrieval (ventral mPFC (vmPFC)) [192]. Furthermore, the decline of extinction in trace fear conditioning [193], spatial learning [194], and auditory threat conditioning which correlated with impaired synaptic plasticity in the BLA [195] has been reported in aged animals. Recent studies have also demonstrated deficits in context-dependent delayed extinction of fear memory in healthy aged humans [196] and worsened performance in visual fear extinction in those with mild cognitive impairment and early stage Alzheimer’s disease [197], as well as in elderly patients with depression [198], compared to healthy aged controls. Alterations in prefrontal neuronal excitability such as increased excitability of the PL and decreased excitability of IL during aging has been suggested to account for, at least in part, the decline of extinction in the aged [193]. Thus, such treatments may improve extinction learning in the aging population as well as disorders such as post-traumatic stress disorder (PTSD) which have been shown to expedite age-related cognitive decline [199] and contribute to the risk of developing dementia [151].

Interestingly, the distributed networks of memories or internal models described here underlying threat conditioning, its extinction, and SOC bear much resemblance to the predictive concepts or schema thought to underlie emotions and their related disorders [5,6,7]. The circuitry supporting threat processing described here likely contributes to such schema, perhaps particularly those underlying emotions such as fear and anxiety [7]. In fact, aberrations of the circuitry involved in threat conditioning and its extinction have been shown to exist in certain psychiatric conditions. For instance, PTSD patients show heightened amygdala activity during the acquisition of threat conditioning [200], as well as functional changes to the vmPFC [201] and impaired extinction [202]. Furthermore, higher-order forms of learning may mimic certain aspects of such pathologies. For instance, SOC may serve as a useful model of panic disorder [157]. In this case, contextual cues may come to be associated with whatever process triggered panic initially, leading to agoraphobia. Here, contextual cues may drive activation of the greater panic state. Similarly, PTSD may depend upon a process by which trauma-related cues come to function as unconditioned stimuli to which further neutral stimuli can be associated, thus broadening and maintaining trauma-related schema [203]. However, although aberrations of basic threat processing may contribute to such disorders, it is important to note that the schema underlying such disorders likely involve additional cognitions such as dysfunctional beliefs which thus need to be addressed if dysfunctional schema are to be dealt with adequately [6]. Failure to address such dysfunctional beliefs may leave fear and anxiety schema susceptible to relapse through reactivation, much like the effect of initial threat conditioning context in the renewal effect. The aim of techniques such as cognitive reappraisal, often used in conjunction with exposure therapy, is to address such beliefs [6,204]. Interestingly, reappraisal is thought to rely on medial and or lateral PFC circuitry [205], and thus may exert its influence in part by dampening the threat processing which may contribute to the maintenance of dysfunctional schema. Thus, a better understanding of the forms of learning discussed in this review may ultimately inform our understanding of, and approach to, disorders related to fear and anxiety.

## 6. Conclusions

The basolateral amygdala exists within a network of structures supporting threat processing. Importantly, aberrations of these processes likely contribute to the etiology of various psychiatric conditions related to fear and anxiety. The schema underlying emotional disorders are not limited to the circuitry described here. As such, dysfunction at either level—the basic circuitry described here or the circuitry underlying higher cognitions such as beliefs—would presumably influence the other, ultimately leading to the maintenance of dysfunctional schema. Thus, our approach to psychiatric conditions dependent on such schema would benefit from a better understanding of each level of processing, as well as how dysfunction may arise within them. Here, we have reviewed the interactions of the BLA within a network of structures for low-level forms of threat processing. Evidence that neuromodulators such as NE and DA serve as important prediction error signals for these forms of learning, as well as evidence regarding their mechanisms of action in these forms of learning, is accumulating. Future work is needed to improve our understanding of how such systems may be leveraged to better interfere with or facilitate the learning processes described here for therapeutic benefit. Evidence for the involvement of the primary and associational sensory cortices in threat conditioning points to the possibility of extinction and SOC processes in the sensory cortices, presenting new avenues for the study of these forms of learning.

## Data Availability

Not applicable.
